# Mechanistic Roles of Matrilin-2 and Klotho in Modulating the Inflammatory Activity of Human Aortic Valve Cells

**DOI:** 10.3390/cells9020385

**Published:** 2020-02-07

**Authors:** Erlinda The, Qingzhou Yao, Peijian Zhang, Yufeng Zhai, Lihua Ao, David A. Fullerton, Xianzhong Meng

**Affiliations:** Department of Surgery, University of Colorado Denver, Aurora, CO 80045, USA; erlinda.the@cuanschutz.edu (E.T.); Qingzhou.yao@cuanschutz.edu (Q.Y.); peijian.zhang@cuanschutz.edu (P.Z.); yufeng.zhai@cuanschutz.edu (Y.Z.); lihua.ao@cuanschutz.edu (L.A.); david.fullerton@cuanschutz.edu (D.A.F.)

**Keywords:** inflammation, NF-κB, PKR, aortic valve, Klotho

## Abstract

Background: Calcific aortic valve disease (CAVD) is a chronic inflammatory disease. Soluble extracellular matrix (ECM) proteins can act as damage-associated molecular patterns and may induce valvular inflammation. Matrilin-2 is an ECM protein and has been found to elevate the pro-osteogenic activity in human aortic valve interstitial cells (AVICs). Klotho, an anti-aging protein, appears to have anti-inflammatory properties. The effect of matrilin-2 and Klotho on AVIC inflammatory responses remains unclear. Methods and Results: Isolated human AVICs were exposed to matrilin*-2*. Soluble matrilin-2 induced the production of ICAM-1, MCP-1, and IL-6. It also induced protein kinase R (PKR) activation via Toll-like receptor (TLR) 2 and 4. Pretreatment with PKR inhibitors inhibited NF-κB activation and inflammatory mediator production induced by matrilin-2. Further, recombinant Klotho suppressed PKR and NF-κB activation and markedly reduced the production of inflammatory mediators in human AVICs exposed to matrilin-2. Conclusions: This study revealed that soluble matrilin-2 upregulates AVIC inflammatory activity via activation of the TLR-PKR-NF-κB pathway and that Klotho is potent to suppress AVIC inflammatory responses to a soluble ECM protein through inhibiting PKR. These novel findings indicate that soluble matrilin-2 may accelerate the progression of CAVD by inducing valvular inflammation and that Klotho has the potential to suppress valvular inflammation.

## 1. Introduction

Calcific aortic valve disease (CAVD) is one of the most common heart valve diseases in the elderly [1]. CAVD manifests clinically as aortic sclerosis or, more severely, as aortic stenosis [2]. Currently, there is no effective pharmacotherapy treatment for CAVD [3]; the only viable treatment option is surgical or interventional valve replacement. These interventions are costly and have limitations due to complications of anticoagulation therapy and the short lifespan of prosthetic valves [4].

For decades, CAVD was viewed as a “mechanical” effect of aging and considered a passive disease [5]. Recent evidence suggests that CAVD is the result of a chronic inflammatory process that promotes aortic valve fibrosis and calcification [6,7]. Innate and adaptive immune responses are involved in CAVD pathobiology [8]. Many studies have reported the pivotal role of inflammation in the initiation and propagation phases of CAVD [9,10]. Diseased aortic valves are found to have higher levels of proinflammatory mediators and inflammatory cell infiltration [11]. It is believed that once inflammatory cells are activated, they will release proinflammatory mediators that subsequently modulate valvular fibrogenic and osteogenic activities [12].

Aortic valve interstitial cells (AVICs) are a heterogeneous population of mostly quiescent fibroblasts that play a crucial role in maintaining valvular homeostasis [13]. Previous studies have found that AVICs play a major role in valvular inflammation [14,15] and contribute to the pathogenesis of CAVD [16,17,18]. Our previous studies have demonstrated that human AVICs express functional TLR2 and TLR4, and activation of these receptors induces the expression of proinflammatory and pro-osteogenic mediators [19,20]. However, little is known about the role of endogenous inducers of AVIC inflammatory response. The inflammatory response of AVICs to damage-associated molecular patterns (DAMPs) may also play a role in the progression of CAVD [21]. *G*rowing evidence indicates several molecules that are normally sequestered in the ECM can function in their soluble form as DAMPs [22]. This occurs when they are proteolytically released from the ECM following tissue stress or injury, initiating an innate immune response to activate and maintain the valvular inflammatory state [23,24,25].

Matrilins are oligomeric extracellular adaptor proteins of the ECM involved in the formation of both collagen-dependent and collagen-independent filamentous networks [26]. Matrilin-2 is expressed in a variety of tissues [27,28,29,30] and can be released from the ECM during tissue injury, becoming available in its soluble form. Changes in matrilin-2 expression have been implicated in some inflammatory diseases [31,32]. We have recently reported that soluble forms of matrilin-2 can upregulate the pro-osteogenic activity in human AVICs via TLR2 and TLR4 [33]. To date, the impact of soluble matrilin-2 on human AVIC production of inflammatory mediators is unknown.

Klotho is an anti-aging protein with anti-inflammatory properties and is expressed primarily in the kidneys [34,35]. Deficiency of Klotho promotes renal inflammation and is also associated with chronic obstructive pulmonary disease [36,37]. Previous studies by our group found that aging-related endotoxemic cardiac dysfunction is associated with relative Klotho deficiency and that treatment with recombinant Klotho suppresses the inflammatory response and improves cardiac function in aging endotoxemic mice [38]. In addition, we found that Klotho levels are lower in calcified human aortic valves [39]. Currently, the effect and mechanism of action of Klotho in modulating the inflammatory response of human AVICs remain unknown.

We hypothesized that soluble matrilin-2 is proinflammatory in human AVICs and that Klotho suppresses the inflammatory response to matrilin-2. The purpose of this study is to determine: (1) whether matrilin-2 induces the production of inflammatory mediators in human AVICs, (2) the mechanism by which matrilin-2 exerts an effect on human AVICs, and (3) whether Klotho suppresses the inflammatory response in human AVICs.

## 2. Materials and Methods

### 2.1. Chemicals and Reagents

Antibodies against ICAM-1 were obtained from Santa Cruz Biotechnology (Santa Cruz, CA, USA). Antibodies against phosphorylated and total NF-κB p65 were obtained from Cell Signaling Technology (Beverly, MA, USA). Antibodies against phosphorylated PKR T446, total PKR, Klotho, and β-actin were obtained from Abcam (Cambridge, MA, USA). Neutralizing antibodies against TLR2 (IMG-416E) and TLR4 (IMG-417E) were obtained from Novus Biologicals (Centennial, CO, USA). Medium 199 was obtained from Thermo Fisher Scientific (Waltham, MA, USA). Recombinant human matrilin-2 and Klotho proteins (expressed by mouse myeloma cell line; endotoxin free) were obtained from R&D Systems (Minneapolis, MN, USA). C_13_H_8_N_4_OS was obtained from MilliporeSigma (Burlington, MA, USA). Pam3CSK4 was obtained from InvivoGen (San Diego, CA, USA). Lipopolysaccharide (LPS, Escherichia coli 0111:B4), Bay11-7082, 2-Aminopurine, collagenase and other reagents were obtained from Sigma-Aldrich (St. Louis, MO, USA).

### 2.2. Isolation and Culture of Human AVICs

The studies were approved by the Institutional Review Board of University of Colorado and performed in accordance with the Declaration of Helsinki 1964 and revision 2013. Written informed consent was obtained from all aortic valve donors prior to their inclusion in this study. Normal tricuspid aortic valves were collected from explanted hearts of heart-transplant recipients at the University of Colorado Hospital.

Normal aortic valve leaflets were excised and washed in 1X phosphate-buffered saline (PBS, Thermo Fisher Scientific, Waltham, MA, USA). The leaflets were then incubated with collagenase solution (type I, 1.0 mg/mL) at 37 °C for 30 min to remove endothelial cells. The undigested tissue was further treated with a fresh solution of 1.0 mg/mL collagenase for 4–6 h at 37 °C to isolate AVICs. After vortexing and aspirating repeatedly to break up the tissue mass, AVICs suspension were centrifuged at 1000 rpm for 10 min. The resulting pellet was resuspended, and cells were cultured in M199 growth medium supplemented with 10% fetal bovine serum, 100 U/mL penicillin, and 100 µg/mL streptomycin in an incubator with 5% CO_2_ at 37 °C. The medium was replaced every 3 days throughout the growth and experimental periods. Human AVICs (cells of passage 3–6, 80–90% confluence) were used for the experiments. All experiments were repeated using AVICs isolated from eight different donor valves.

Human AVICs were stimulated with recombinant matrilin-2 (2.0 μg/mL) for different time periods to determine the impact of soluble matrilin-2 on cellular inflammatory activity. We have found in a previous study that this concentration of matrilin-2 is capable of activating NF-κB [33]. To evaluate the roles of NF-κB and PKR in mediating the effect of matrilin-2, cells were treated with NF-κB inhibitor Bay11-7082 (10 μM) and PKR inhibitors 2-AP (1.0 mM) or C_13_H_8_N_4_OS (0.5 μM) one hour before matrilin-2 treatment. The concentrations of these pharmacological reagents were determined in reference of published studies [33,40]. To assess the effect of Klotho on AVIC inflammatory activity, recombinant Klotho was applied as a pretreatment in a concentration (0.5 μg/mL) used in our previous study [39].

### 2.3. Immunoblotting

Immunoblotting was applied to analyze ICAM-1, phosphorylated and total NF-κB, phosphorylated and total PKR, and Klotho. AVICs were washed with PBS and lysed in 2x Laemmli sample buffer (Biorad, Hercules, CA, USA). Samples were separated by 4–20% SDS-PAGE gels and transferred to nitrocellulose membranes. The membranes were blocked with 5% skim milk solution for 1 h at room temperature, and then incubated with primary antibodies (1:200 to 1:1000 volume dilution) overnight at 4 °C, followed by incubation with a secondary antibody for 1 h. Membranes were developed using the enhanced chemiluminescence system. β-actin was analyzed for normalization of protein loading. In phosphorylation assay, total NF-κB and total PKR were used for normalization. Quantification of band density was performed using the ImageLab software of the Bio-Rad.

### 2.4. Quantitative qPCR

Total RNA was extracted from AVICs using the TRI Reagent (Sigma-Aldrich, St. Louis, MO, USA), quantified on a NanoDrop spectrophotometer (Thermo Fisher Scientific, Waltham, MA, USA), and reverse transcribed into cDNA with iScript cDNA Synthesis Kit (Bio-Rad, Hercules, CA, USA). Gene-specific primers used for the qPCR analysis were: human-KL Forward: 5′-CTCGGGTACCTGGTGGC-3′; human-KL Reverse: 5′-ACACCTGACCTCCCTGAGTG-3′. Quantitative qPCR was performed using a LightCycler ^®^ 96 Real-Time PCR machine (Roche Diagnostic, Basel, Switzerland) using SYBR Green Supermix (Bio-Rad, Hercules, CA, USA). The expression of mRNA was calculated as the relative ratio to human GAPDH mRNA, with all data presented as fold change against control.

### 2.5. Immunofluorescence Staining

Immunofluorescence detection and localization of PKR and NF-κB proteins in AVICs were performed. Cells were seeded in 8-well chamber slides and treated with recombinant matrlin2 (2 μg/mL) or left untreated for indicated time periods. PKR inhibitors were added to AVICs 1 h prior to the addition of recombinant matrilin-2. After treatment, cells were rinsed briefly with PBS, permeabilized with 70% methanol 30% acetone mixture, then fixed with 4% paraformaldehyde for 15 min at room temperature. Non-specific binding of the antibodies was blocked with 10% goat serum for 30 min at room temperature. Cells were then incubated with an antibody against PKR (1:100 dilution) or NF-κB p65 (1:50 dilution) at 4 °C overnight. Following a final wash with PBS, cells were incubated with Cy3-conjugated secondary antibody for 2 h at room temperature. The nuclei were counterstained with 4′,6-Diamidino-2-phenylindole (DAPI). Microscopy was performed using a Leica CTR5500 digital microscope (Leica Mikroskopie und Systeme GmbH, Wetzlar, Germany).

### 2.6. ELISA

Commercial ELISA kits (R & D Systems, Minneapolis, MN, USA) were utilized to quantify MCP-1, TNF-α, IL-1β and IL-6 in the supernatant of AVIC culture. Samples and standards were prepared according to manufacturer’s instructions. Absorbance of standards and samples were determined spectrophotometrically at 450 nm, using a microplate reader (Biotek, Winooski, VT, USA). Results were plotted against the linear portion of the standard curve.

### 2.7. Statistical Analysis

Statistical analyses were performed using Prism Software (GraphPad, San Jose, CA, USA). Data are presented as mean ± standard error (SE). The statistical significance of the difference between two sets of data was assessed using student t-test. One-way analysis of variance with Bonferroni’s post hoc test was used to analyze differences between multiple groups. *P* value ≤ 0.05 was considered to be significant.

## 3. Results

### 3.1. Matrilin-2 Induces the Inflammatory Responses in Human AVICs

**Figure 1:**
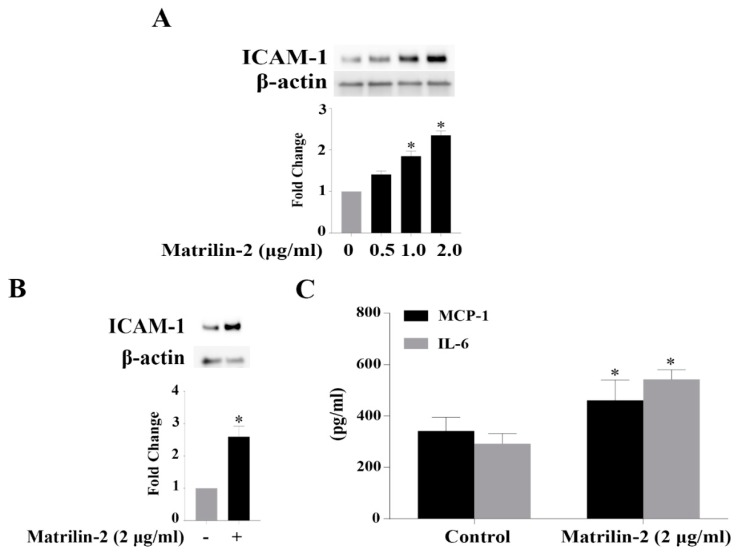
Matrilin-2 induces the inflammatory responses in human aortic valve interstitial cells (AVICs). Human AVICs were stimulated with different concentrations of recombinant matrilin-2 for 48 h. (**A**) Recombinant matrilin-2 has a dose-dependent effect on ICAM-1 expression in human AVICs. (**B**) Recombinant matrilin-2 (2.0 μg/mL) increases ICAM-1 levels. (**C**) Recombinant matrilin-2 promotes the release of MCP-1 and IL-6. Values are means ± SE. n = 5 experiments using distinct cell isolates; * *P* < 0.05 vs. control.

We determined the effect of matrilin-2 on the inflammatory activity in human AVICs. Protein levels of ICAM-1 were examined after stimulation with increasing concentrations of matrilin-2 (0, 0.5, 1.0, and 2.0 μg/mL) for 48 h. Figure 1A shows that cellular ICAM-1 levels increased in a dose-dependent fashion and the highest level was observed in cells exposed to 2.0 μg/mL of matrilin-2 (Figure 1B). We then applied ELISA assay to analyze inflammatory cytokines secreted by human AVICs after an exposure to matrilin-2 (2.0 μg/mL). As shown in Figure 1C, AVICs released greater levels of MCP-1 and IL-6 following stimulation with matrilin-2 for 48 h. These data demonstrate that soluble matrilin-2 is potent to induce the inflammatory responses in human AVICs.

### 3.2. Matrilin-2 Activates PKR and NF-κB in Human AVICs

**Figure 2:**
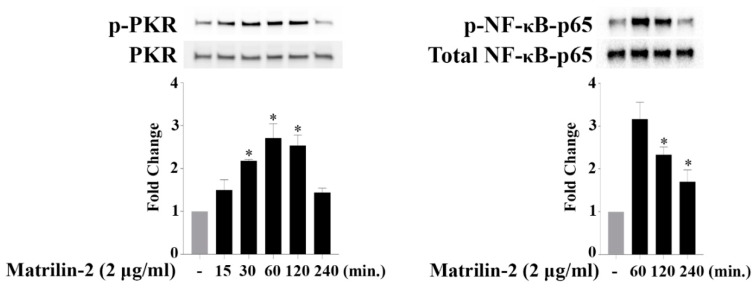
Matrilin-2 activates PKR and NF-κB in human AVICs. Human AVICs were stimulated with recombinant matrilin-2 for varied durations. Stimulation with recombinant matrilin-2 resulted in increased levels of phospho-PKR and phospho-NF-κB. Values are means ± SE. n = 5 experiments using distinct cell isolates; * *P* < 0.05 vs. control.

To test the hypothesis that PKR mediates AVIC inflammatory responses to soluble ECM protein, we examined whether soluble matrilin-2 activates PKR in human AVICs. As shown in Figure 2, PKR phosphorylation gradually increased and peaked at 1 h after matrilin-2 stimulation, then returned to baseline after 4 h. We utilized immunofluorescence staining to localize PKR in human AVICs. Following matrilin-2 stimulation, no intranuclear translocation of PKR was observed ( Supplementary Figure S1). Our findings suggest that PKR is activated when human AVICs are exposed to soluble matrilin-2 and that PKR may not directly induce the expression of inflammatory mediators. Then, we examined NF-κB activation following matrilin-2 stimulation since our previous study found that soluble matrilin-2 induces NF-κB activation in human AVICs. As shown in Figure 2, phosphorylation of NF-κB p65 was markedly increased after 1 h of treatment with matrilin-2 and activation of NF-κB was temporarily correlated with PKR activation. Taken together, our results demonstrate that soluble matrilin-2 activates both PKR and NF-κB in human AVICs.

### 3.3. The PKR-NF-κB Pathway Mediates Matrilin-2–induced Inflammatory Responses

**Figure 3:**
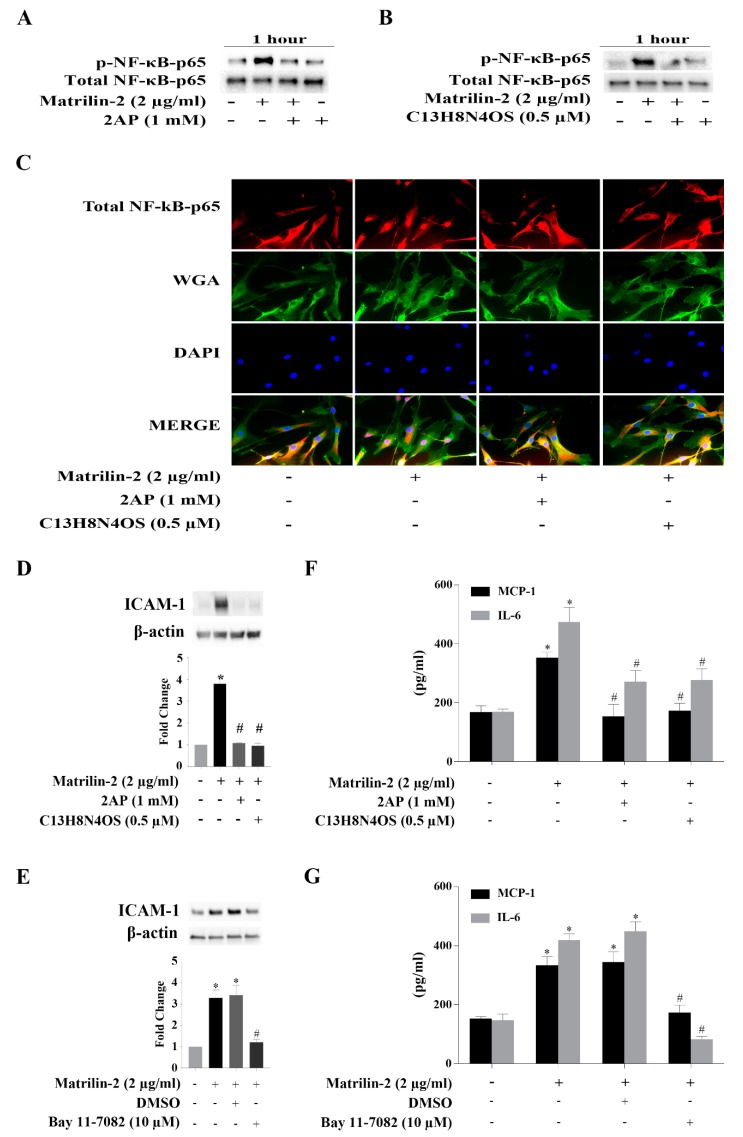
Both PKR and NF-κB are critical for AVIC inflammatory responses induced by matrilin-2, and PKR is responsible for NF-κB activation. Human AVICs were treated with PKR inhibitors (C_13_H_8_N_4_OS and 2-AP) or NF-κB inhibitor (Bay 11-7082) for 1 h or left untreated, followed by stimulation with recombinant matrilin-2 for 1 h or 48 h. (**A**,**B**) Inhibition of PKR suppressed NF-κB phosphorylation. (**C**) Nuclear translocation of NF-κB was inhibited by PKR inhibitors. Representative images of immunofluorescence staining show NF-κB (red) in human AVICs. Alexa 488–tagged wheat germ agglutinin (WGA) was applied to outline plasma membrane (green). DAPI (4′,6-diamidino-2-phenylindole) was applied for nuclei counterstaining (blue). Original magnification, ×40 objective. (**D**,**E**) Inhibition of PKR or NF-κB markedly reduced ICAM-1 production following matrilin-2 stimulation. (**F**,**G**) PKR and NF-κB inhibitors markedly reduced MCP-1 and IL-6 release following stimulation with matrilin-2. Values are means ± SE. n = 5 experiments using distinct cell isolates; * *P* < 0.05 vs. control; # *P* < 0.05 vs. matrilin-2 alone.

To determine whether there is an interaction between PKR and NF-κB in human AVICs following matrilin-2 stimulation, we assessed the effect of pharmacological inhibition of PKR. The induction of PKR activation by matrilin-2 in human AVICs was inhibited by either of the two PKR inhibitors (Supplementary Figure S2), and inhibition of PKR suppressed soluble matrilin-2-induced NF-κB activation (Figure 3A,B). In addition, immunofluorescence staining results confirmed the inhibitory effect of PKR inhibitors on matrilin-2-induced NF-κB p65 translocation to the nucleus (Figure 3C).

We then examined whether soluble matrilin-2 induces the inflammatory responses via the PKR-NF-κB signaling pathway. Human AVICs were treated with 2-AP, C_13_H_8_N_4_OS or Bay11-7082 for 1 h or left untreated before stimulation with matrilin-2 for 48 h. Inhibition of PKR or NF-κB suppressed the expression of ICAM-1 (Figure 3D,E), and the production of MCP-1 and IL-6 production (Figure 3F,G). These findings reveal that PKR is upstream of NF-κB and that the PKR-NF-κB signaling pathway mediates the inflammatory responses to soluble matrilin-2 in human AVICs.

### 3.4. TLR2 and TLR4 Activate PKR to Induce the Inflammatory Responses in Human AVICs

**Figure 4:**
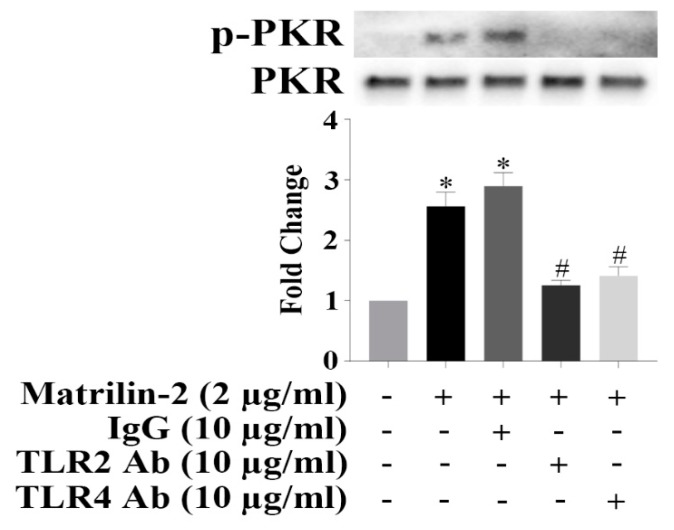
Both toll-like receptor (TLR) 2 and 4 mediate matrilin-2-induced PKR activation in human AVICs. Human AVICs were treated with TLR2-neutralizing antibody or TLR4-neutralizing antibody for 1 h or left untreated, followed by stimulation with recombinant matrilin-2. Neutralization either TLR2 or TLR4 attenuated matrilin-2-induced PKR phosphorylation. Values are means ± SE. n = 3 separate experiments; * *P* < 0.05 vs. control; # *P* < 0.05 vs. matrilin-2 alone.

Because TLR2 and TLR4 play an important role in mediating pro-inflammatory and pro-osteogenic responses, we examined whether TLR2 and TLR4 mediate PKR activation induced by matrilin-2 in human AVICs. As shown in Supplementary Figure S3, TLR2 agonist PAM3CSK4 and TLR4 agonist LPS induced PKR activation in human AVICs. We treated human AVICs with and without neutralizing antibodies against TLR2 or TLR4 prior to matrilin-2 stimulation. The result in Figure 4 shows that soluble matrilin-2-induced PKR activation was inhibited by neutralizing either TLR2 or TLR4. These results indicate that simultaneous engagement of TLR2 and TLR4 is crucial for the activation of PKR by matrilin-2 in human AVICs.

### 3.5. Klotho Suppresses Matrilin-2-induced Inflammatory Responses in Human AVICs

**Figure 5:**
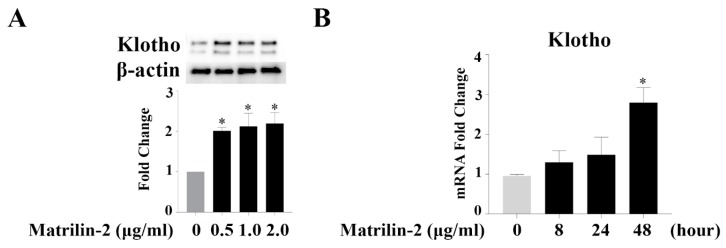
Human AVICs express Klotho. (**A**) Klotho protein is present in human AVICs and is upregulated by matrilin-2 stimulation for 48 h. (**B**) Klotho mRNA is detectable in human AVICs and increases in a time-dependent manner following matrilin-2 stimulation. Values are means ± SE. n = 3 separate experiments; * *P* < 0.05 vs. control.

To confirm the expression of Klotho in human AVICs, protein and mRNA levels were examined by Western blot and RT-PCR. We observed that Klotho protein and mRNA are present in human AVICs (Figure 5A,B). Interestingly, the levels of Klotho mRNA and protein increased after matrilin-2 stimulation.

**Figure 6:**
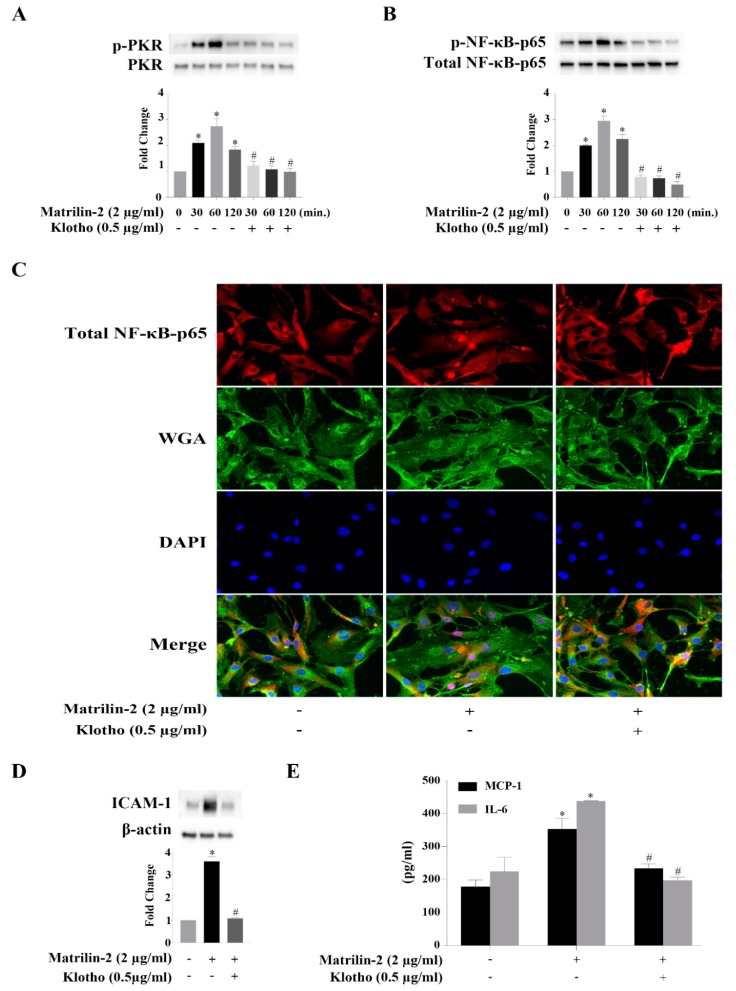
Klotho suppresses matrilin-2-induced inflammatory responses in human AVICs via inhibition of the PKR-NF-κB cascade. Human AVICs were treated with recombinant Klotho (0.5 μg/mL) for 2 h or left untreated, followed by stimulation with matrilin-2. (**A**) Recombinant Klotho suppressed matrilin-2-induced PKR activation. (**B**) Recombinant Klotho attenuated soluble matrilin-2-induced NF-κB activation. Values are means ± SE. n = 3 separate experiment; * *P* < 0.05 vs. control; # *P* < 0.05 vs. the same time point of matrilin-2 alone. (**C**) Klotho inhibited matrilin*-2*-induced NF-κB nuclear translocation. Representative images of immunofluorescence staining show NF-κB (red) in human AVICs. Alexa 488-tagged WGA (green) was applied to outline plasma membrane. DAPI (blue) was applied for nuclear counterstaining. Original magnification, ×40 objective. (**D**) Klotho attenuated matrilin-2-induced ICAM-1 expression. (**E**) Klotho reduced MCP-1 and IL-6 production induced by matrilin-2. Values are means ± SE. n = 5 separate experiments using distinct cell isolates; * *P* < 0.05 vs. control; # *P* < 0.05 vs. matrilin-2 alone.

We treated human AVICs with recombinant Klotho 2 h prior to matrilin-2 stimulation to determine its effect on the inflammatory responses to matrilin-2. Western blotting examination showed that pretreatment of human AVICs with recombinant Klotho inhibited matrilin-2-induced activation of PKR (Figure 6A) and NF-κB (Figure 6B). In addition, Klotho prevented NF-κB intranuclear translocation induced by matrilin-2 (Figure 6C). Moreover, Klotho pretreatment suppressed the upregulation of ICAM-1 (Figure 6D), MCP-1 and IL-6 (Figure 6E). Therefore, Klotho exerts an anti-inflammatory effect in human AVICs, and it suppresses matrilin-2-induced inflammatory responses through inhibition of PKR and NF-κB activation.

## 4. Discussion

CAVD is one of the most prevalent cardiovascular diseases in the elderly people. This disease progresses to symptomatic severe aortic stenosis in many cases, necessitating costly aortic valve replacements. The pathobiology of CAVD involves chronic inflammation [41]. AVICs are the predominant cells in aortic valve leaflets and play an important role in maintaining valvular homeostasis. In the pathological condition of CAVD, however, AVICs synthesize and secret ECM proteins, cytokines and growth factors and thus become key cells involved in valvular inflammation [42]. However, the mechanism by which AVICs adapt a proinflammatory phenotype remains unclear [43].

In the present study, we demonstrate: (1) soluble ECM protein matrilin-2 induces the inflammatory responses in human AVICs; (2) a TLRs-PKR-NF-κB signaling cascade mediates the inflammatory responses of human AVICs to soluble matrilin-2; (3) human AVICs express Klotho; (4) Klotho is potent to suppress AVIC inflammatory responses to soluble matrilin-2 by inhibiting the PKR-NF-κB signaling pathway. These observations provide new insights into the mechanism by which soluble ECM proteins induce valvular inflammation, which plays a mechanistic role in CAVD progression. Further, this study demonstrates that Klotho has the potential for suppression of valvular inflammation.

In our previous study, we observed that biglycan, an ECM protein, can induce the inflammatory responses in human AVICs [44]. Accumulating evidence suggests that soluble ECM proteins can function as DAMPs to induce the inflammatory responses [22], and DAMPs have attracted attention for their potential role in CAVD progression [21]. In this regard, our previous studies demonstrated that soluble matrilin-2 promotes the pro-osteogenic activity in human AVICs by a TLR-dependent mechanism [33]. However, the impact of soluble matrilin-2 on AVIC inflammatory responses is unclear. In the present study, we observed that soluble matrilin-2 upregulates the production of ICAM-1, MCP-1 and IL-6 in human AVICs. ICAM-1, MCP-1, and IL-6 are essential for leukocyte migration and adhesion to tissue [45,46]. This is an important event in tissue inflammation associated with different inflammatory diseases including CAVD [47]. Thus, our results indicate that soluble matrilin-2 is one of the soluble ECM proteins that function as DAMPs to activate innate immunity in human AVICs.

PKR was originally identified as an antiviral protein and has long been known to be involved in the innate immunity [48]. PKR has been found to play a critical role in the activation of inflammasomes [40]. In addition, recent study has reported that PKR mediates the inflammatory responses to hyperosmotic stress in mouse embryonic fibroblasts cells [49]. The role of PKR in the regulation of inflammatory responses in human AVICs has never been studied. Our results show that soluble matrilin-2 activates PKR, and PKR in human AVICs is linked to NF-κB. Results presented herein implicate a novel role of PKR, as a critical upstream factor in mediating the NF-κB-dependent inflammatory responses to soluble matrilin-2 in human AVICs.

NF-κB is a well-known regulator of inflammatory responses through inducing transcription of proinflammatory genes [50]. NF-κB is retained in an inactive form in the cytoplasm in most cell types [51]. When it is activated, NF-κB translocates to the nucleus and plays a critical role in activating the inflammatory signaling network [52]. Our previous study found that NF-κB activation contributes to the inflammatory responses in human AVICs [44].

In this study, we observed that NF-κB signaling is activated in human AVICs by soluble matrilin-2, which is consistent with our previous findings [33]. In addition, we found that NF-κB activation is associated with PKR phosphorylation. Based on this observation, we hypothesized that PKR regulates NF-κB activation to induce the inflammatory responses in human AVICs. We applied two specific PKR inhibitors (2-AP and C_13_H_8_N_4_OS) to determine the link between PKR and NF-κB in human AVICs. Notably, inhibition of PKR with either 2-AP or C_13_H_8_N_4_OS abrogated soluble matrilin-2-induced NF-κB activation. More importantly, inhibition of PKR prevented NF-κB intranuclear translocation in human AVICs exposed to soluble matrilin-2. These results support the notion that soluble matrilin-2 induces the NF-κB-dependent inflammatory responses in human AVICs through activation of PKR.

Multiple proinflammatory signaling pathways can be activated by TLR-2 and TLR-4 activation [53,54]. In this study, we observed that TLR2 and TLR4 mediate PKR activation in human AVICs in response to soluble matrilin-2 as neutralization of either TLR2 or TLR4 abrogated PKR activation. In addition, we found that stimulation of human AVICs with TLR2 ligand Pam3csk4 and TLR4 ligand LPS also activates PKR in human AVICs. These observations are consistent with our previous report that soluble matrilin-2 induces the osteogenic responses in human AVICs through a mechanism involving both TLR2 and TLR4 [33]. Collectively, our findings demonstrate that soluble matrilin-2 engages the TLRs-PKR-NF-κB signaling cascade to induce the inflammatory responses in human AVICs. It is noteworthy that both inflammatory and osteogenic responses in human AVICs can be induced by matrilin-2. One possibility is that the NF-κB signaling activates a mechanism that mediates the expression of both inflammatory and osteogenic mediators. Alternatively, the inflammatory mediators mediated by NF-κB may upregulate the production of osteogenic factors. Future studies are needed to address this notion. Nevertheless, the effect of soluble ECM proteins on human AVIC inflammatory and osteogenic responses indicate that they may play a role in promoting valvular inflammation and calcification associated with CAVD progression.

Klotho is a membrane protein, and a soluble form of this protein is identified in the blood, cerebral fluid and urine, presumably exhibiting pleiotropic actions [55]. Klotho was originally identified as an anti-aging protein mainly expressed in renal tubule epithelial cells [56]. Interestingly, Klotho mRNA and protein are detected in human AVICs in the present study. Expression of Klotho is upregulated moderately in human AVICs after exposure to soluble matrilin-2. Importantly, our results show that treatment with recombinant Klotho inhibits PKR to suppress the inflammatory responses in human AVICs exposed to matrilin-2. Thus, our findings demonstrate that human AVICs express Klotho, which help maintain the homeostasis of human AVICs exposed to a pro-inflammatory stimulus. In this regard, several studies find that Klotho has an anti-inflammatory function [36,57]. We recently observed that Klotho suppresses myocardial inflammation and preserves cardiac function in aging endotoxemic mice [38]. Interestingly, Klotho levels are lower in calcified aortic valves from patients with advanced CAVD [39]. The relative Klotho insufficiency in diseased aortic valves may be attributed to aging, the disease pathobiology, or both. Given the anti-inflammatory effect of Klotho, it is reasonable to propose that Klotho has therapeutic potential for suppression of valvular inflammation associated with CAVD progression.

One of the limitations in this study is the relatively small sample size. While all experiments were repeated using cell isolates from different donors, this small group of normal aortic valves used for this study cannot provide information regarding the influence of clinical factors, such as age, gender, and heart disease, on AVIC response to a pro-inflammatory stimulation. As CAVD is a chronic inflammatory condition, the findings that endogenous proteins modulate human AVIC inflammatory activity are significant for improving the understanding of mechanisms underlying aortic valve inflammation associated with CAVD progression. However, translation of the in vitro findings to in vivo conditions should be taken with caution.

## 5. Conclusions

This study demonstrates that soluble matrilin-2 activates the TLRs-PKR-NF-κB signaling pathway to induce inflammatory responses in human AVICs. Klotho suppresses AVIC inflammatory responses to soluble matrilin-2 via inhibition of PKR. These novel findings indicate that soluble matrilin-2 may contribute to the pathobiology of CAVD by upregulation of AVIC inflammatory activity. Further, this study highlights the role of Klotho in suppressing valvular inflammation via inhibition of the pro-inflammatory signaling mechanisms. We speculate that insufficiency of this anti-aging protein may have a role in the pathogenesis of CAVD, an aging-related valvular heart disease, and that recombinant Klotho may have therapeutic potential to prevent CAVD progression.

## Supplementary Materials

The following are available online at https://www.mdpi.com/2073-4409/9/2/385/s1, Figure S1: PKR is localized in the cytosol of human AVICs. Human AVICs were treated with recombinant matrilin-2 for 30 min to 1 h. Representative images of immunofluorescence staining show PKR (red) in human AVICs. Alexa 488-tagged WGA (green) was applied to outline plasma membrane. DAPI (blue) was applied for nuclear counterstaining. Original magnification, ×40 objective, Figure S2: PKR inhibitors abolish matrilin-2-induced PKR phosphorylation in human AVICs. Human AVICs were treated with C13H8N4OS or 2-AP for 1 h or left untreated, followed by stimulation with matrilin-2 (2.0 μg/mL) for 1 h, Figure S3: Stimulation of TLR2 or TLR4 with a specific agonist induces PKR phosphorylation. Human AVICs were treated with TLR2 agonist Pam3csk4 or TLR4 agonist lipopolysaccharide (LPS) for 15 to 240 min.
