# Supplementary material for: Mechanistic Roles of Matrilin-2 and Klotho in Modulating the Inflammatory Activity of Human Aortic Valve Cells
Source: Cells. 2020 Feb 7;9(2):385. doi: 10.3390/cells9020385 (PMC7072362; doi:10.3390/cells9020385)
Supplement: Supplementary file 1 [file cells-09-00385-s001.pdf]

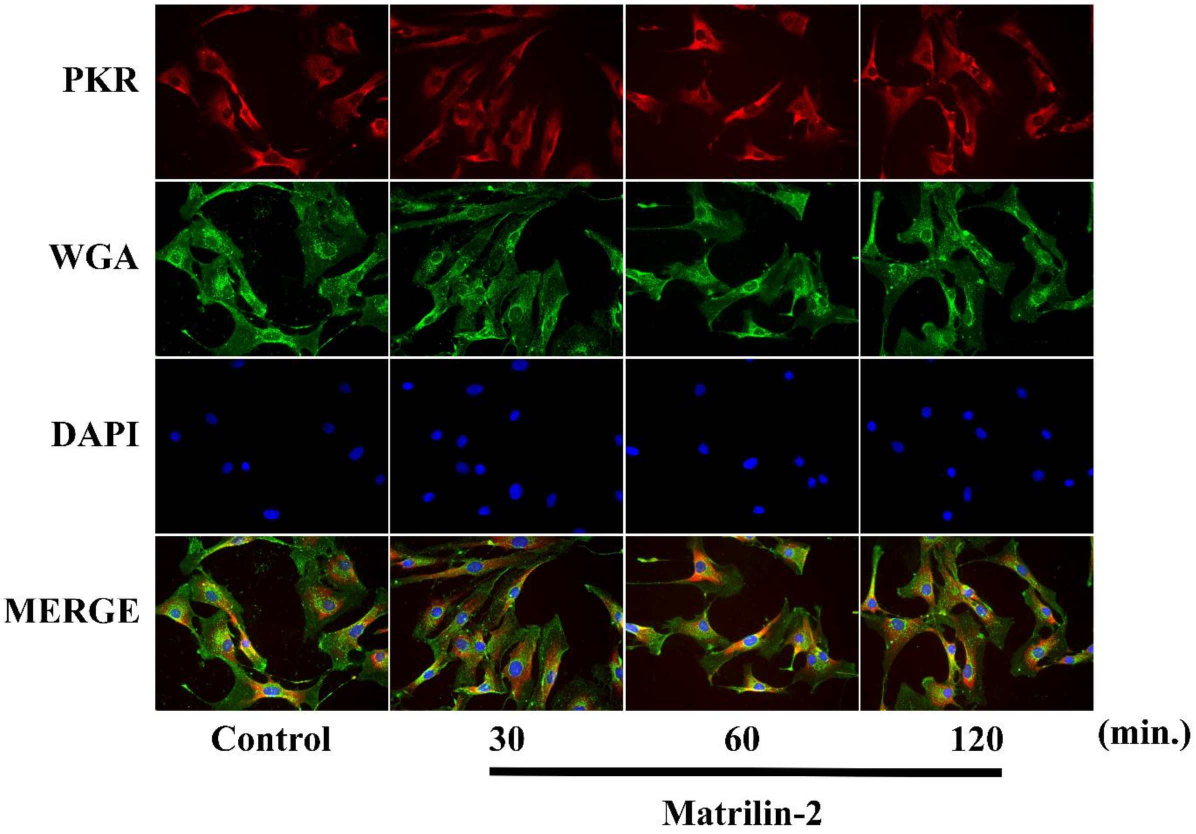

Supplementary Figure 1. PKR is localized in the cytosol of human AVICs. Human AVICs were treated with recombinant matrilin-2 for 30 mins to 1h. Representative images of immunofluorescence staining show PKR (red) in human AVICs. Alexa 488-tagged WGA (green) was applied to outline plasma membrane. DAPI (blue) was applied for nuclear counterstaining. Original magnification, ×40 objective.

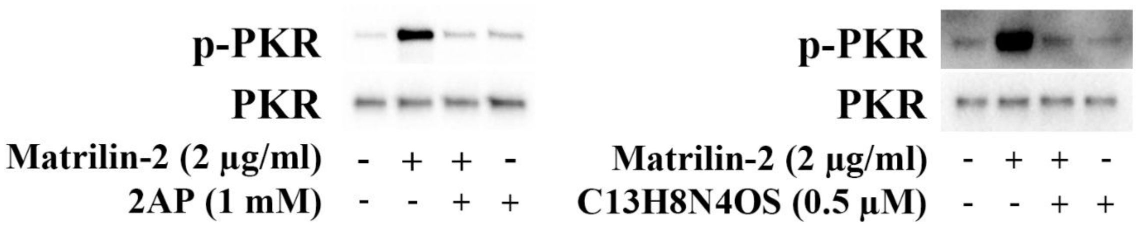

Supplementary Figure 2. PKR inhibitors abolish matrilin-2-induced PKR phosphorylation in human AVICs. Human AVICs were treated with $C_{13}H_8N_4OS$ or 2-AP for 1h or left untreated, followed by stimulation with matrilin-2 (2.0 μg/ml) for 1h.

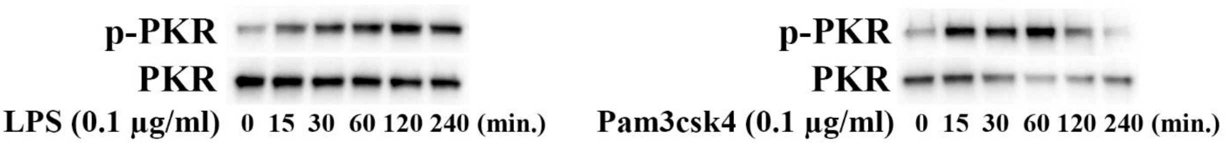

Supplementary Figure 3. Stimulation of TLR2 or TLR4 with a specific agonist induces PKR phosphorylation. Human AVICs were treated with TLR2 agonist Pam3csk4 or TLR4 agonist lipopolysaccharide (LPS) for 15 to 240 min.
